# Intellectual property licensing of therapeutics during the COVID-19 crisis: lessons learnt for pandemic preparedness and response

**DOI:** 10.1186/s12992-024-01057-5

**Published:** 2024-07-02

**Authors:** Tiwadayo Braimoh, Esteban Burrone, Charles Gore, Pushpa Vijayaraghavan

**Affiliations:** 1 Strategy, Policy and Market Access, Medicines Patent Pool, Geneva, Switzerland; 2 Management, Medicines Patent Pool, Geneva, Switzerland; 3 Governance Board, Medicines Patent Pool, Geneva, Switzerland

**Keywords:** Intellectual property, Voluntary licensing, Access, Therapeutics, COVID-19, Generic, LMICs

## Abstract

During the COVID-19 pandemic, intellectual property licensing through bilateral agreements and the Medicines Patent Pool were used to facilitate access to new COVID-19 therapeutics in low- and middle-income countries (LMICs). The lessons learnt from the application of the model to COVID-19 could be relevant for preparedness and response to future pandemics and other health emergencies.

The speed at which affordable versions of a new product are available in LMICs is key to the realization of the potential global impact of the product. When initiated early in the research and development life cycle, licensing could facilitate rapid development of generic versions of innovative products in LMICs during a pandemic. The pre-selection of qualified manufacturers, for instance building on the existing network of generic manufacturers engaged during the COVID-19 pandemic, the sharing of know-how and the quick provision of critical inputs such as reference listed drugs (RLDs) could also result in significant time saved. It is important to find a good balance between speed and quality. Necessary quality assurance terms need to be included in licensing agreements, and the potentials of the new World Health Organization Listed Authority mechanism could be explored to promote expedited regulatory reviews and timely access to safe and quality-assured products.

The number, capacity, and geographical distribution of licensed companies and the transparency of licensing agreements have implications for the sufficiency of supply, affordability, and supply security. To foster competition and support supply security, licenses should be non-exclusive. There is also a need to put modalities in place to de-risk the development of critical pandemic therapeutics, particularly where generic product development is initiated before the innovator product is proven to be effective and approved. IP licensing and technology transfer can be effective tools to improve the diversification of manufacturing and need to be explored for regional manufacturing for accelerated access at scale in in LMICs and supply security in future pandemics.

## Background

International policy attention has been focused on pandemic prevention, preparedness, and response (PPPR) to avoid a repeat of some of the failures in the response to the COVID-19 crisis in the future. A central issue in the PPPR discussions is equitable access to medical countermeasures, often with a spotlight on access to intellectual property (IP), technology, and know-how. These have been a critical part of discussions under the World Health Organization (WHO) processes to amend the International Health Regulations (IHR) and develop a pandemic agreement [1, 2]. Lessons learnt from the application of voluntary IP licensing of COVID-19 therapeutics could be relevant for preparedness and response to future pandemics. An IP license is a legal authority on IP rights granted by an IP rights holder to a third-party, for instance to enable the manufacture and supply of a patented product, typically under specified terms and conditions [3–5]. Other forms of IP licenses which are not the focus of this paper include compulsory and government-use licenses, where a national authority permits an entity other than the patent holder to produce, import, sell or use a patent-protected product [6]. Without licenses or other relevant interventions, patented products cannot legally be produced and/or sold as lower priced generics before patent expiry in the jurisdictions of the patents [6]. The need to strengthen PPPR, including a recognition of the value of building on lessons learned from IP sharing mechanisms was recognized in the Political Declaration of the United Nations General Assembly High-level Meeting on PPPR [7].

During the COVID-19 pandemic, license agreements were signed bilaterally between business entities and also through the Medicines Patent Pool (MPP) to enable access to therapeutics in low- and middle-income countries (LMICs) [8, 9]. At least 90 agreements for COVID-19 therapeutics were signed, about two-thirds of which were with MPP [10]. MPP is a public health organization that works to facilitate affordable access to new health technologies in LMICs through licensing and technology transfer. Its aim is to help reduce global health inequity by increasing the availability of innovative pharmaceutical products for populations in LMICs. Established by Unitaid in 2010 with an initial focus on expanding access to HIV medicines, MPP’s mandate has progressively expanded to cover other disease areas and health technologies. Shadlen [11] identified the "virtues" of MPP licenses from a global health perspective, highlighting their public-health focus, transparency, and non-exclusivity and the benefits and impact of MPP licenses have been reported in other publications [5, 12, 13]

The bilateral COVID-19 therapeutic license agreements include the deals between Gilead Sciences and nine generic companies for remdesivir; Merck, Sharp & Dohme’s (MSD) agreement with nine manufacturers for molnupiravir; and Eli Lilly’s agreement with eight manufacturers for the supply of baricitinib in India [10]. In certain instances such as for tocilizumab and ritonavir, patent holders decided not to assert their patent rights [14, 15]. MPP’s agreements were with MSD and 27 generic manufacturers for molnupiravir; Pfizer and 36 generic manufacturers for nirmatrelvir co-packaged with ritonavir; and Shionogi and seven generic manufacturers for ensitrelvir [16]. Being the first orally administered COVID-19 therapeutics that could be used on an out-patient basis to be approved for emergency use, [17–19] molnupiravir, nirmatrelvir/ritonavir and ensitrelvir were of particular interest to many stakeholders [20]. The full license agreements between MPP and the respective innovators were also made publicly available [21].

### Licensing of oral COVID-19 therapeutics

The first step towards the licensing of COVID-19 therapeutics by MPP was the identification of priority COVID-19 therapeutics in the research and development (R&D) pipeline for which licensing and technology transfer could facilitate access in LMICs. This was done under the auspices of the therapeutics pillar of the Access to COVID-19 Tools Accelerator (ACT-A) and the focus was on oral antivirals in late-stage development. MPP then reached out to the innovator pharmaceutical companies to explore licenses that would support access to the promising investigational treatments in LMICs. Its negotiations with MSD, Pfizer, and Shionogi resulted in the signing of transparent public health-oriented license agreements for molnupiravir, nirmatrelvir/ritonavir and ensitrelvir respectively. Following the execution of the licenses, open calls for expressions of interest were announced, inviting qualified manufacturers to apply. A total of 207 manufacturers from 37 countries applied for a license and following a rigorous process of assessment of applications, MPP signed sublicense agreements with selected generic manufacturers based in 16 countries across all continents. The head licenses were signed prior to regulatory authorizations of the innovator products and the sublicensees were announced thereafter following the expression of interest process. As is the case with all MPP’s licenses, the license and sublicense agreements were published on the MPP website [16].

#### License territory and other key terms

A total of 119 LMICs representing 56% of the world’s population are covered by at least one MPP COVID-19 therapeutic license. These include 106 LMICs for molnupiravir, 95 for nirmatrelvir/ritonavir, and 117 for ensitrelvir. The agreement does not prevent sales outside the licensed territory where doing so would not infringe on any patents and if the licensees are not utilizing the optional licensed know-how. All three licenses were royalty free during the period that COVID-19 was classified as a Public Health Emergency of International Concern (PHEIC) by the WHO. Afterwards, each sublicense is royalty-bearing at 5% of net sales for supply in the public sector and 10% for sales in the private sector. In the case of nirmatrelvir/ritonavir and ensitrelvir the licenses remain royalty-free in low-income countries even after the end of the PHEIC [16].

#### Product development

With innovators’ support, facilitated by MPP, sublicensees undertook active pharmaceutical ingredient (API) and formulation development, bioequivalence studies, stability studies, dossier compilation, and regulatory filing. MPP sublicensees are comprised of manufacturers that produce only APIs, only finished pharmaceutical product (FPP), and those that are vertically integrated and manufacture both. The support given included the provision of confidential technical packages for know-how for API and FPP development and supply of the reference listed drugs (RLDs). RLDs are innovator products that are previously approved by relevant regulatory authorities, with which generic versions are compared in a test to demonstrate bioequivalence as a part of the requirement for the regulatory approval of the generic product. Taking the know-how package was optional for the companies and 18% of molnupiravir and 80% of nirmatrelvir/ritonavir sublicensees took it. As mentioned earlier, sublicensees that take the technical know-how packages can only supply within the license territory (those that do not may under certain circumstances supply beyond the licence territory, e.g. when/if there are no patents on the product in the relevant countries. The decisions to access or not to access the packages may have been based on factors including manufacturer capabilities, timing of the license and the relative complexities of the development and manufacture of the products. Innovators’ support was also critical to access RLDs, as they were not obtainable from the market. Despite considerable efforts from all parties (innovators, MPP and generic manufacturers) to accelerate delivery of the RLD to all licensees, there were still challenges on the path to delivering the RLDs, including customs bottlenecks. The timeframe for product development varied between companies as sublicensees started off from different capacity and know-how baselines, with some initiating the process even before licensing.

#### Regulatory review and market entry

Under the license, for quality assurance purposes, licensees were required to obtain approval (or emergency use authorization) from a Stringent Regulatory Authority (SRA) or the WHO Prequalification (PQ) Programme, except for time-bound conditional waivers granted for molnupiravir and ensitrelvir. In addition, companies needed to obtain the necessary regulatory authorizations in countries, where required. The fastest MPP sublicensee submission to WHO PQ was for nirmatrelvir/ritonavir. The finished dosage form (FDF) dossier was filed four months after the announcement of the sub-license agreement and WHO PQ was secured after nine months (or one year from the approval of the innovator product). Before the pandemic, it had taken at best three to four years to achieve WHO PQ for a generic version of a new medicine from the date in which the innovator had obtained first approval. HIV medicines dolutegravir and tenofovir alafenamide, for example, took three years and four months and four years and two months respectively [22–24]. Regulatory processes took longer periods prior to the pandemic, but various other factors, including the lengthy process of developing the generic product and demonstrating its bioequivalence contributed to the delays.

In the case of molnupiravir, in light of the aforementioned waiver, licensees that had met certain conditions, such as authorization from the national regulatory authorities of the country of manufacture and sale, were able to begin supplying directly earlier, before obtaining WHO PQ or SRA approval. This, however, did not enable procurement and supply via the international agencies, which required SRA or WHO PQ approvals (or emergency use authorizations). Nevertheless, subsequent WHO PQ paved the way for the supply of COVID-19 oral therapeutics in the license territory via the international agencies. While at least 16 LMICs have benefitted from access to the products, demand has generally remained low. Reasons for this are varied and include timing, low diagnostic rates in many LMICs, and challenges in the roll-out and use of the products. By the time the products were developed, vaccines had been available for over a year, and the peak of the pandemic had passed. With earlier product development and regulatory approval and accelerated global access, the utility of therapeutics could be different in future pandemics.

### Lessons learnt for pandemic preparedness and response

Based on MPP’s experience with the application of the voluntary licensing model to COVID-19, important lessons can be learnt for pandemic preparedness and response. The lessons learnt include what would be needed to achieve rapid availability of affordable quality-assured therapeutics in sufficient quantities and as needed across the world upon the declaration of a health emergency. In other words, the objective of IP licensing for pandemic response should be to support speed in access, affordability, quality, volumes, and supply security. The points below reflect on how to achieve this. It should be noted that the discovery and development of effective innovative therapeutics is a pre-requisite for access-oriented licensing to be possible, and efforts are already taking place to support discovery and development of medicines with activity against pathogens of pandemic potential, with a view to having phase-2 ready compounds across viral classes [25]. The latter issue, however, is not within the scope of this paper. The focus here is on supporting access to such medicines through licensing, with a particular focus on equitable access in LMICs.

#### Once a new therapeutic of value in a pandemic is proven to be effective, the speed at which affordable versions of the product become available in LMICs is key to the realization of the potential global impact of the product

The licensing of promising therapeutics early in the process of innovative product development as well as the sharing of technical know-how, and in some cases starting materials, could be important enablers of rapid access in LMICs during a pandemic. Granting licenses early enables the development of generic versions of novel products by LMIC manufacturers to begin while the innovator product is still in clinical development. If the product proves effective, suppliers are already advancing product development and the lag to market can be shortened. As seen with nirmatrelvir/ritonavir, the sharing of technical know-how can contribute, at times quite significantly, to accelerating development. The transfer of know-how and technology in some cases can also contribute to smoother and faster regulatory review for the generic product. With relevant incentives and supportive conditions such as the inclusion of relevant access terms in R&D funding agreements and early discussions with innovative product developers during the pre-pandemic period, the stage for the sharing of intellectual property and know-how could be set from the early phases of R&D and licensees’ processes could go in parallel while innovators lead the way in product development.

The prior identification of qualified manufacturers that would be capable and ready to start product development when it is needed in a pandemic could also result in significant time saved. As proposed by the G20 health ministers in India in 2023, the existing MPP network of generic manufacturers engaged in the context of COVID-19 could be a good starting point and be further developed and built upon to make it fit-for-purpose as a manufacturing network for equitable access to therapeutics during future pandemics [26]. Having a geographically distributed network of manufacturers with the necessary capabilities across different product types (e.g. tablets, injectables, biologics) committed to contributing to pandemic response, and developing standard operating procedures ahead of a pandemic, could help reduce potential delays from manufacturer selection during a pandemic and enable swifter implementation.

Another important factor for the rapid development of licensed products and the shortening of the gap in the time to market between innovator products and their generic versions is the quick provision of critical inputs such as RLDs for bioequivalence studies, and where necessary, key starting materials to generic/biosimilar manufacturers. As with COVID-19, under emergency situations, innovator products that could be used as RLDs may not be commercially available and there may not be any alternative to direct provision of the RLD by innovators. It would be important to define streamlined mechanisms for ensuring timely access to RLDs ahead of a pandemic, including for example, addressing key challenges such as customs and trade-related bottlenecks that could significantly delay the supply of RLD across borders. In addition to the crucial need for know-how, access to starting materials could obviate the need for generic/biosimilar manufacturers to start product development from scratch, especially for products for which reverse engineering may be difficult, expensive or time-consuming, such as biologics.

#### While speed is of essence in a pandemic, it is important to ensure that it is not achieved at the expense of quality. Therefore, there is a need for expedited regulatory mechanisms that accelerate availability while providing the necessary quality assurance

Quality assurance provisions are an important part of all licensing agreements and the global public health community may need to further explore expedited regulatory approaches or pathways to shorten the time to regulatory approvals and availability of the licensed products. In some instances, during the COVID-19 pandemic, licensed manufacturers secured approvals (or emergency use listings) from national regulatory authorities in LMICs far ahead of obtaining WHO PQ or SRA approvals which are often required by global health agencies.

This could create a situation whereby a product approved by a national regulatory authority cannot be supplied in that country if the quality assurance mechanisms required under the license and by global health entities for procurements have not been met. The ongoing transition from SRA to WHO Listed Authorities (WLA) with the prospect that regulatory authorities in LMICs included in licenses may one day qualify as WLAs, and the adoption of such standard by international procurers (e.g. the Global Fund), may contribute to overcoming this challenge in the future [27, 28]. Moreover, the use of mechanisms for regulatory cooperation and reliance that accelerate national regulatory procedures for products already assessed elsewhere provide additional pathways for expedited access to licensed therapeutics during a pandemic that could be further strengthened [27, 29].

It may also be important to consider clinical development strategies by innovators that can contribute to earlier access in the future in LMICs. For example, the inclusion of clinical trials sites in certain LMICs where the products may be most needed or where key licensees may be based can contribute to faster regulatory reviews of the generic product in such countries and contribute to expedited access.

#### Building an adequate supply base for therapeutics could contribute to affordability and supply security and additional market shaping and health systems interventions may be critical for products to reach those who need them during a pandemic

To foster competition, it is important to ensure that a sufficient number of manufacturers receive a license, but the uncertainties of pandemics could make the determination of volumes that would be needed and accurate estimation of the market potential of new therapeutics challenging. Too few licensees could be a risk for undersupply if there is a surge in cases and could mean less competition and higher prices; too many licensees could diminish the viability of the market and sustainability of supplies. When demand is not realized as was the case with COVID-19, licensees may have no adequate returns on their investments and the appetite to continue to invest in pandemic products for current or future pandemics. Despite the challenges, the estimation of volumes that could be needed during a health emergency by relevant international health agencies would be helpful to support licensing efforts.

In addition to demand predictability, structural problems including fragmented and under-funded markets in certain LMICs are a steep challenge for suppliers. There is a need to put measures in place to address hurdles and disincentives on the path to access. It is important to de-risk the development of critical pandemic therapeutics by generic manufacturers, particularly where product development is initiated before a treatment has proven to be effective or when future demand estimation is particularly challenging. This could be through targeted push and pull interventions, including for example, payments tied to product development milestones and/or advance market commitments. The creation of funded local or regional procurement and/or stockpiling mechanisms before a pandemic could be crucial to indicate demand and mitigate possible risks to access to therapeutics during health emergencies.

The transparency of licensing agreements creates a level playing field and supports competitiveness, in addition to being a good practice that aligns well with global calls for greater transparency in the markets for pharmaceuticals [30].

#### Licensing can also be a tool to improve the diversification of manufacturing and supply security for future pandemics and respond to calls for local/regional manufacturing of key health products

Local/regional manufacturing is important to help reduce the excessive dependence of some countries or regions on imported products and the associated risks. During the COVID-19 pandemic, certain countries where some of the world's largest producers and exporters of health products are located introduced restrictive measures on exports or closed their country borders leading to extended procurement lead-times and supply shortages in many other countries [31]. To allow for equitable manufacturing capacities across the world, the manufacturing networks established for the COVID-19 therapeutics could be further diversified. This may need to go hand in hand with support for the further development of manufacturing capacity. Alternatively, or in addition, companies from regions that are historically under-represented in licensee manufacturer maps could be offered the possibility of being included in the network of manufacturers for pandemic response if certain conditions are met, for example the attainment of Good Manufacturing Practice certification. This could be an incentive to companies which may otherwise self-disqualify or would need further investments to meet the necessary requirements. It could also signal to governments and stakeholders that support local manufacturer capacities that a given company could be suitable to target for support. During the COVID-19 pandemic, only a few sublicensees from certain countries demonstrated the capacity to deliver rapidly, underscoring the need for interventions to support local/regional manufacturers to ensure they are able to deliver at the scale and timelines that are compatible with a pandemic response. Such support, while important for pandemic response, needs to have a broader focus to ensure sustainability.

MPP’s agreements with innovators are typically the result of efforts at striking a delicate balance between the commercial interests of patent holders, market viability and sustainability for the generic manufacturers and most importantly equity and public health. Being voluntary in nature, the licenses typically focus on countries that are outside the key commercial markets of the innovators. This is particularly true for licenses with the pharmaceutical industry on promising new products and may be quite different from licenses on early-stage technologies from public research organizations, such as those negotiated by the COVID Technology Access Pool. Higher royalties in certain (comparatively wealthier) countries or public–private market segmentation have at times been used to enable broader geographical scope for some of MPP licenses [12]. Further incentives or policies may need to be explored that could support the application of a licensing strategy in additional countries in future pandemics and other health emergencies.

## Conclusion

IP licensing and technology transfer can be effective mechanisms for enabling access to therapeutics at scale in an accelerated manner during a pandemic, and existing models such as MPP and its expertise, track record and established networks could be leveraged to achieve important PPPR milestones. The work and investment of the broader stakeholder groups (e.g. governments, regional and global inter-governmental organizations, the private sector, regulators, funders) as well as collaborative engagement among actors across all levels would be key to build and nurture a manufacturing ecosystem that is ready to deliver for regional and global health security. While this paper is focused mainly on small molecules, licensing and technology transfer is equally applicable to biotherapeutics and vaccines. If rapid and equitable access to therapeutics is to be realized for LMICs in future pandemics, the need for early access to IP, technology, know-how, and critical inputs; pre-selected network of manufacturers; capacity development within the network; de-risking of certain critical investments by manufacturers; expedited regulatory reviews and approvals; regional manufacturing, and other relevant interventions and processes would need to be addressed, mostly at pre-pandemic times. Several articles in the draft texts of the pandemic agreement submitted to the 77^th^ World Health Assembly by the WHO contain proposed provisions which, if adopted, could significantly enable some of these recommendations.

## Data Availability

No datasets were generated or analysed during the current study.
